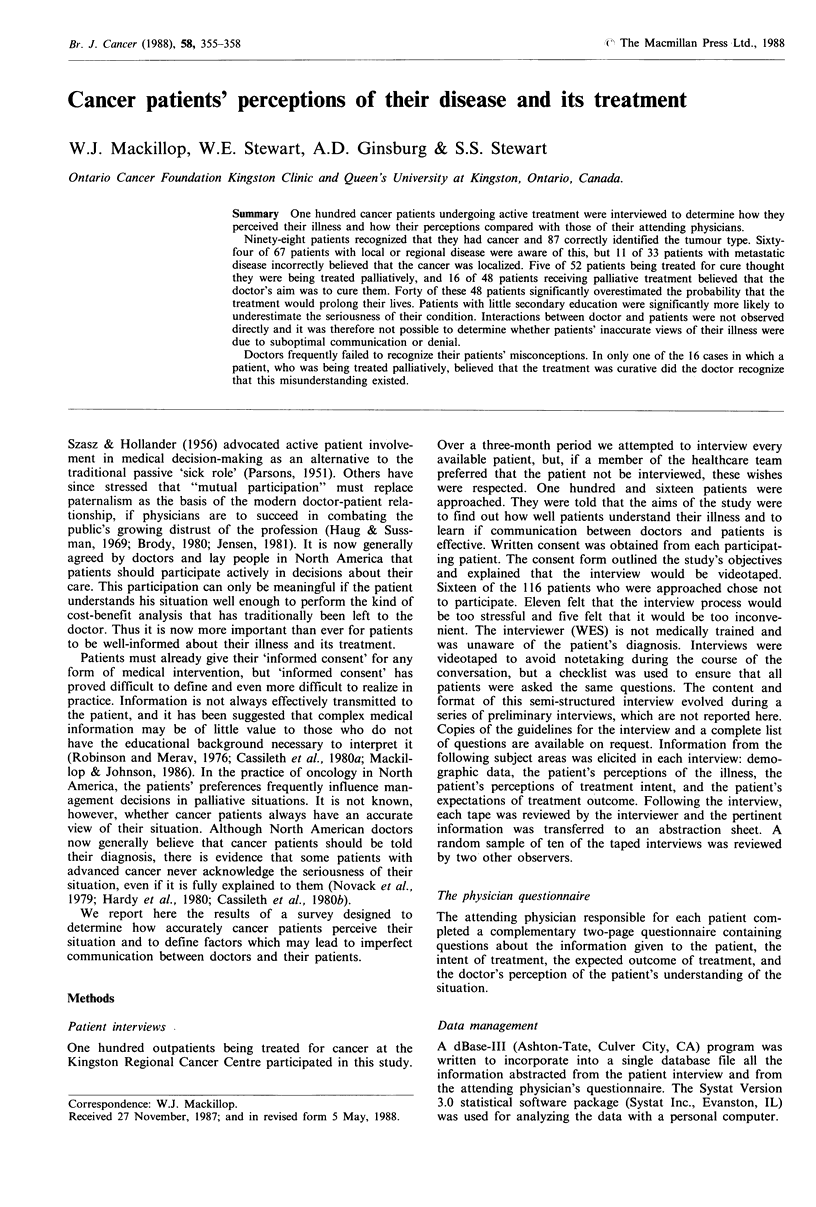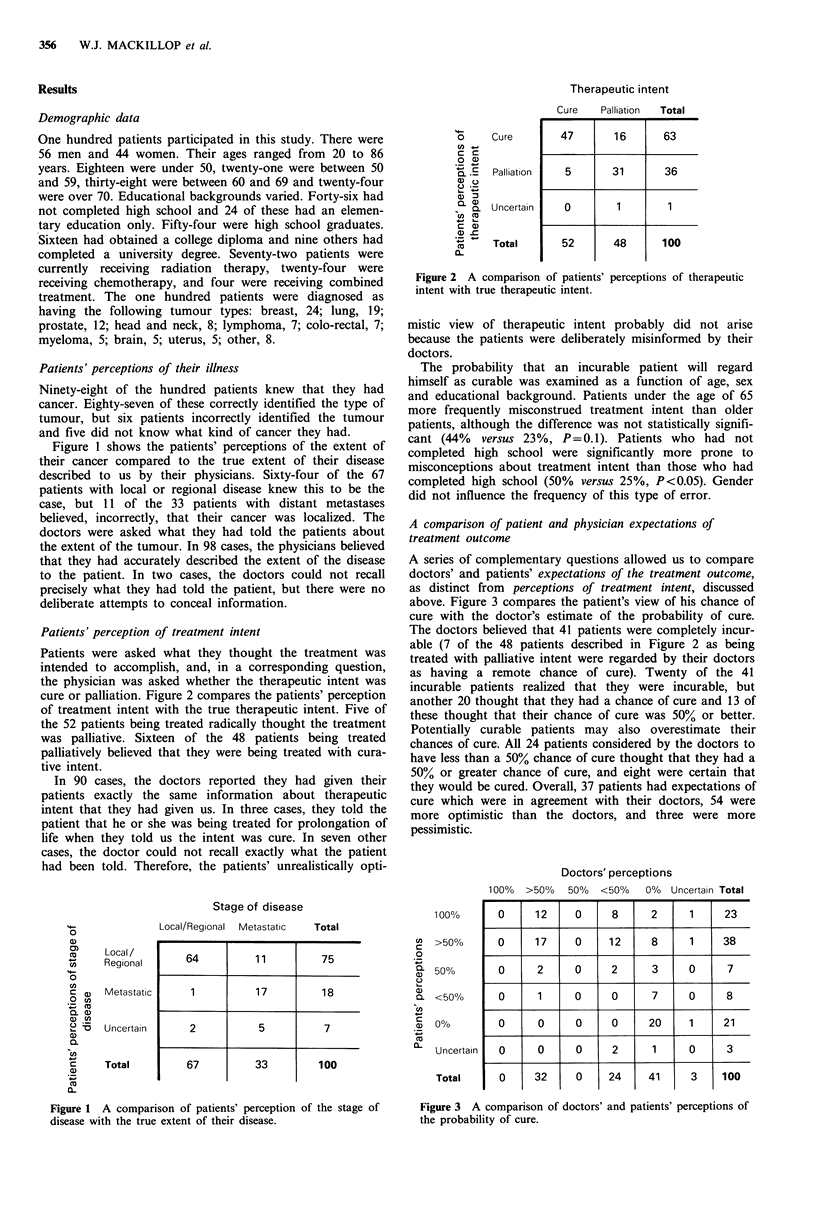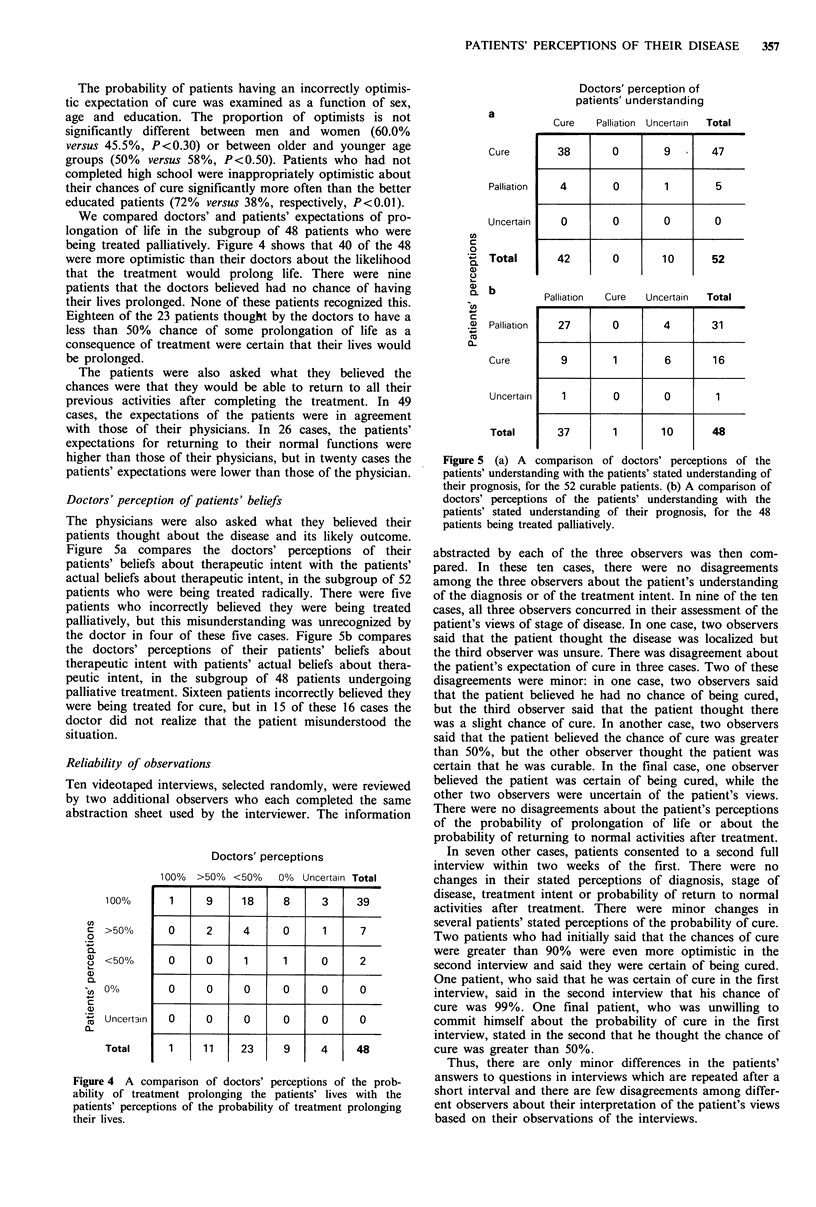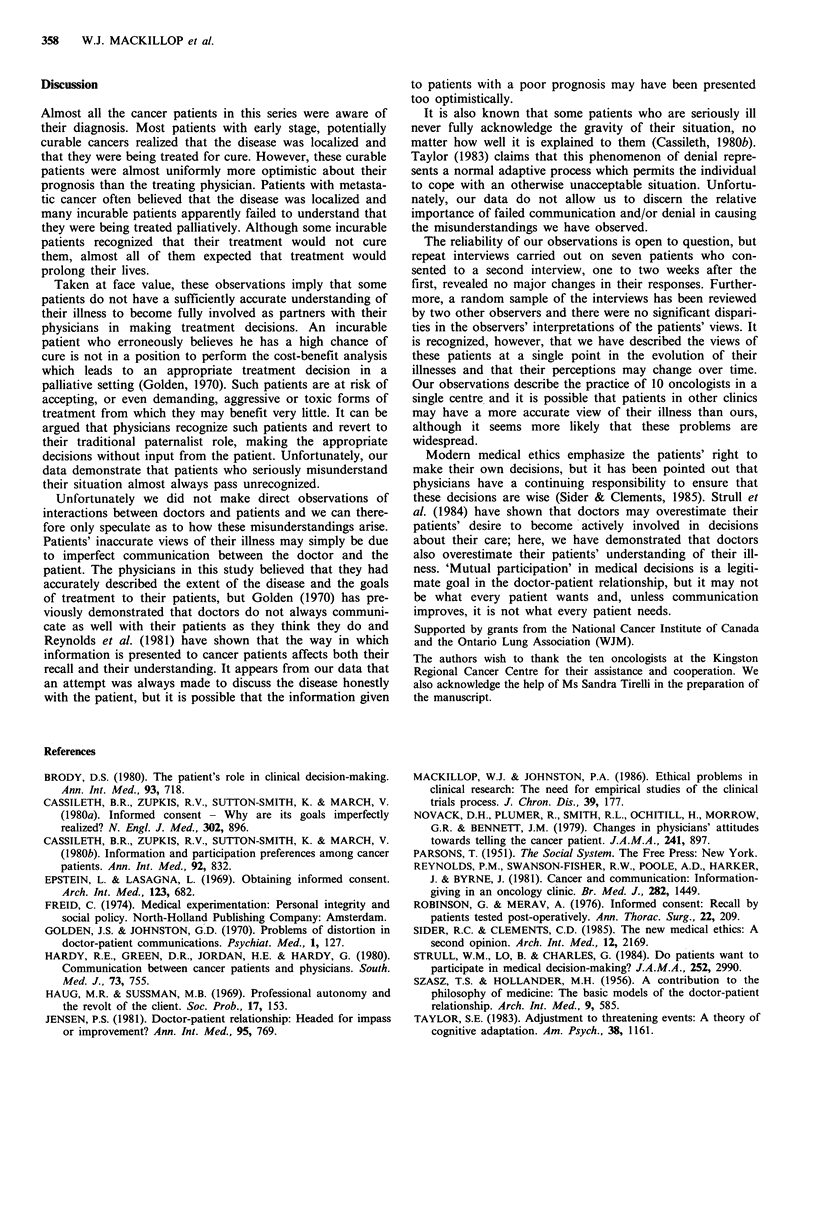# Cancer patients' perceptions of their disease and its treatment.

**DOI:** 10.1038/bjc.1988.218

**Published:** 1988-09

**Authors:** W. J. Mackillop, W. E. Stewart, A. D. Ginsburg, S. S. Stewart

**Affiliations:** Ontario Cancer Foundation Kingston Clinic, Canada.

## Abstract

One hundred cancer patients undergoing active treatment were interviewed to determine how they perceived their illness and how their perceptions compared with those of their attending physicians. Ninety-eight patients recognized that they had cancer and 87 correctly identified the tumour type. Sixty-four of 67 patients with local or regional disease were aware of this, but 11 of 33 patients with metastatic disease incorrectly believed that the cancer was localized. Five of 52 patients being treated for cure thought they were being treated palliatively, and 16 of 48 patients receiving palliative treatment believed that the doctor's aim was to cure them. Forty of these 48 patients significantly overestimated the probability that the treatment would prolong their lives. Patients with little secondary education were significantly more likely to underestimate the seriousness of their condition. Interactions between doctor and patients were not observed directly and it was therefore not possible to determine whether patients' inaccurate views of their illness were due to suboptimal communication or denial. Doctors frequently failed to recognize their patients' misconceptions. In only one of the 16 cases in which a patient, who was being treated palliatively, believed that the treatment was curative did the doctor recognize that this misunderstanding existed.


Br. J. Cancer (1988), 58, 355–358 © The Macmillan Press Ltd., 1988

### Cancer patients' perceptions of their disease and its treatment

W.J. Mackillop, W.E. Stewart, A.D. Ginsburg & S.S. Stewart

*Ontario Cancer Foundation Kingston Clinic and Queen's University at Kingston, Ontario, Canada.*

**Summary** One hundred cancer patients undergoing active treatment were interviewed to determine how they perceived their illness and how their perceptions compared with those of their attending physicians.

Ninety-eight patients recognized that they had cancer and 87 correctly identified the tumour type. Sixty-four of 67 patients with local or regional disease were aware of this, but 11 of 33 patients with metastatic disease incorrectly believed that the cancer was localized. Five of 52 patients being treated for cure thought they were being treated palliatively, and 16 of 48 patients receiving palliative treatment believed that the doctor's aim was to cure them. Forty of these 48 patients significantly overestimated the probability that the treatment would prolong their lives. Patients with little secondary education were significantly more likely to underestimate the seriousness of their condition. Interactions between doctor and patients were not observed directly and it was therefore not possible to determine whether patients' inaccurate views of their illness were due to suboptimal communication or denial.

Doctors frequently failed to recognize their patients' misconceptions. In only one of the 16 cases in which a patient, who was being treated palliatively, believed that the treatment was curative did the doctor recognize that this misunderstanding existed.

Szasz & Hollander (1956) advocated active patient involvement in medical decision-making as an alternative to the traditional passive 'sick role' (Parsons, 1951). Others have since stressed that "mutual participation" must replace paternalism as the basis of the modern doctor-patient relationship, if physicians are to succeed in combating the public's growing distrust of the profession (Haug & Sussman, 1969; Brody, 1980; Jensen, 1981). It is now generally agreed by doctors and lay people in North America that patients should participate actively in decisions about their care. This participation can only be meaningful if the patient understands his situation well enough to perform the kind of cost-benefit analysis that has traditionally been left to the doctor. Thus it is now more important than ever for patients to be well-informed about their illness and its treatment.

Patients must already give their 'informed consent' for any form of medical intervention, but 'informed consent' has proved difficult to define and even more difficult to realize in practice. Information is not always effectively transmitted to the patient, and it has been suggested that complex medical information may be of little value to those who do not have the educational background necessary to interpret it (Robinson and Merav, 1976; Cassileth *et al.*, 1980*a*; Mackillop & Johnson, 1986). In the practice of oncology in North America, the patients' preferences frequently influence management decisions in palliative situations. It is not known, however, whether cancer patients always have an accurate view of their situation. Although North American doctors now generally believe that cancer patients should be told their diagnosis, there is evidence that some patients with advanced cancer never acknowledge the seriousness of their situation, even if it is fully explained to them (Novack *et al.*, 1979; Hardy *et al.*, 1980; Cassileth *et al.*, 1980*b*).

We report here the results of a survey designed to determine how accurately cancer patients perceive their situation and to define factors which may lead to imperfect communication between doctors and their patients.

### Methods

### *Patient interviews*

One hundred outpatients being treated for cancer at the Kingston Regional Cancer Centre participated in this study. Over a three-month period we attempted to interview every available patient, but, if a member of the healthcare team preferred that the patient not be interviewed, these wishes were respected. One hundred and sixteen patients were approached. They were told that the aims of the study were to find out how well patients understand their illness and to learn if communication between doctors and patients is effective. Written consent was obtained from each participating patient. The consent form outlined the study's objectives and explained that the interview would be videotaped. Sixteen of the 116 patients who were approached chose not to participate. Eleven felt that the interview process would be too stressful and five felt that it would be too inconvenient. The interviewer (WES) is not medically trained and was unaware of the patient's diagnosis. Interviews were videotaped to avoid notetaking during the course of the conversation, but a checklist was used to ensure that all patients were asked the same questions. The content and format of this semi-structured interview evolved during a series of preliminary interviews, which are not reported here. Copies of the guidelines for the interview and a complete list of questions are available on request. Information from the following subject areas was elicited in each interview: demographic data, the patient's perceptions of the illness, the patient's perceptions of treatment intent, and the patient's expectations of treatment outcome. Following the interview, each tape was reviewed by the interviewer and the pertinent information was transferred to an abstraction sheet. A random sample of ten of the taped interviews was reviewed by two other observers.

### *The physician questionnaire*

The attending physician responsible for each patient completed a complementary two-page questionnaire containing questions about the information given to the patient, the intent of treatment, the expected outcome of treatment, and the doctor's perception of the patient's understanding of the situation.

### *Data management*

A dBase-III (Ashton-Tate, Culver City, CA) program was written to incorporate into a single database file all the information abstracted from the patient interview and from the attending physician's questionnaire. The Systat Version 3.0 statistical software package (Systat Inc., Evanston, IL) was used for analyzing the data with a personal computer.

Correspondence: W.J. Mackillop.
Received 27 November, 1987; and in revised form 5 May, 1988.

### Results

### *Demographic data*

One hundred patients participated in this study. There were 56 men and 44 women. Their ages ranged from 20 to 86 years. Eighteen were under 50, twenty-one were between 50 and 59, thirty-eight were between 60 and 69 and twenty-four were over 70. Educational backgrounds varied. Forty-six had not completed high school and 24 of these had an elementary education only. Fifty-four were high school graduates. Sixteen had obtained a college diploma and nine others had completed a university degree. Seventy-two patients were currently receiving radiation therapy, twenty-four were receiving chemotherapy, and four were receiving combined treatment. The one hundred patients were diagnosed as having the following tumour types: breast, 24; lung, 19; prostate, 12; head and neck, 8; lymphoma, 7; colo-rectal, 7; myeloma, 5; brain, 5; uterus, 5; other, 8.

### *Patients' perceptions of their illness*

Ninety-eight of the hundred patients knew that they had cancer. Eighty-seven of these correctly identified the type of tumour, but six patients incorrectly identified the tumour and five did not know what kind of cancer they had.

Figure 1 shows the patients' perceptions of the extent of their cancer compared to the true extent of their disease described to us by their physicians. Sixty-four of the 67 patients with local or regional disease knew this to be the case, but 11 of the 33 patients with distant metastases believed, incorrectly, that their cancer was localized. The doctors were asked what they had told the patients about the extent of the tumour. In 98 cases, the physicians believed that they had accurately described the extent of the disease to the patient. In two cases, the doctors could not recall precisely what they had told the patient, but there were no deliberate attempts to conceal information.

### *Patients' perception of treatment intent*

Patients were asked what they thought the treatment was intended to accomplish, and, in a corresponding question, the physician was asked whether the therapeutic intent was cure or palliation. Figure 2 compares the patients' perception of treatment intent with the true therapeutic intent. Five of the 52 patients being treated radically thought the treatment was palliative. Sixteen of the 48 patients being treated palliatively believed that they were being treated with curative intent.

In 90 cases, the doctors reported they had given their patients exactly the same information about therapeutic intent that they had given us. In three cases, they told the patient that he or she was being treated for prolongation of life when they told us the intent was cure. In seven other cases, the doctor could not recall exactly what the patient had been told. Therefore, the patients' unrealistically optimistic view of therapeutic intent probably did not arise because the patients were deliberately misinformed by their doctors.

| Patients' perceptions of stage of disease | Stage of disease: Local/Regional | Metastatic | Total |
|---|---|---|---|
| Local/ Regional | 64 | 11 | 75 |
| Metastatic | 1 | 17 | 18 |
| Uncertain | 2 | 5 | 7 |
| **Total** | 67 | 33 | **100** |

**Figure 1** A comparison of patients' perception of the stage of disease with the true extent of their disease.

| Patients' perceptions of therapeutic intent | Therapeutic intent: Cure | Palliation | **Total** |
|---|---|---|---|
| Cure | 47 | 16 | 63 |
| Palliation | 5 | 31 | 36 |
| Uncertain | 0 | 1 | 1 |
| **Total** | 52 | 48 | **100** |

**Figure 2** A comparison of patients' perceptions of therapeutic intent with true therapeutic intent.

The probability that an incurable patient will regard himself as curable was examined as a function of age, sex and educational background. Patients under the age of 65 more frequently misconstrued treatment intent than older patients, although the difference was not statistically significant (44% *versus* 23%, $P=0.1$). Patients who had not completed high school were significantly more prone to misconceptions about treatment intent than those who had completed high school (50% *versus* 25%, $P<0.05$). Gender did not influence the frequency of this type of error.

### *A comparison of patient and physician expectations of treatment outcome*

A series of complementary questions allowed us to compare doctors' and patients' *expectations of the treatment outcome*, as distinct from *perceptions of treatment intent*, discussed above. Figure 3 compares the patient's view of his chance of cure with the doctor's estimate of the probability of cure. The doctors believed that 41 patients were completely incurable (7 of the 48 patients described in Figure 2 as being treated with palliative intent were regarded by their doctors as having a remote chance of cure). Twenty of the 41 incurable patients realized that they were incurable, but another 20 thought that they had a chance of cure and 13 of these thought that their chance of cure was 50% or better. Potentially curable patients may also overestimate their chances of cure. All 24 patients considered by the doctors to have less than a 50% chance of cure thought that they had a 50% or greater chance of cure, and eight were certain that they would be cured. Overall, 37 patients had expectations of cure which were in agreement with their doctors, 54 were more optimistic than the doctors, and three were more pessimistic.

| Patients' perceptions | Doctors' perceptions: 100% | >50% | 50% | <50% | 0% | Uncertain | **Total** |
|---|---|---|---|---|---|---|---|
| 100% | 0 | 12 | 0 | 8 | 2 | 1 | 23 |
| >50% | 0 | 17 | 0 | 12 | 8 | 1 | 38 |
| 50% | 0 | 2 | 0 | 2 | 3 | 0 | 7 |
| <50% | 0 | 1 | 0 | 0 | 7 | 0 | 8 |
| 0% | 0 | 0 | 0 | 0 | 20 | 1 | 21 |
| Uncertain | 0 | 0 | 0 | 2 | 1 | 0 | 3 |
| **Total** | 0 | 32 | 0 | 24 | 41 | 3 | **100** |

**Figure 3** A comparison of doctors' and patients' perceptions of the probability of cure.

The probability of patients having an incorrectly optimistic expectation of cure was examined as a function of sex, age and education. The proportion of optimists is not significantly different between men and women (60.0% *versus* 45.5%, $P<0.30$) or between older and younger age groups (50% *versus* 58%, $P<0.50$). Patients who had not completed high school were inappropriately optimistic about their chances of cure significantly more often than the better educated patients (72% *versus* 38%, respectively, $P<0.01$).

We compared doctors' and patients' expectations of prolongation of life in the subgroup of 48 patients who were being treated palliatively. Figure 4 shows that 40 of the 48 were more optimistic than their doctors about the likelihood that the treatment would prolong life. There were nine patients that the doctors believed had no chance of having their lives prolonged. None of these patients recognized this. Eighteen of the 23 patients thought by the doctors to have a less than 50% chance of some prolongation of life as a consequence of treatment were certain that their lives would be prolonged.

The patients were also asked what they believed the chances were that they would be able to return to all their previous activities after completing the treatment. In 49 cases, the expectations of the patients were in agreement with those of their physicians. In 26 cases, the patients' expectations for returning to their normal functions were higher than those of their physicians, but in twenty cases the patients' expectations were lower than those of the physician.

*Doctors' perception of patients' beliefs*

The physicians were also asked what they believed their patients thought about the disease and its likely outcome. Figure 5a compares the doctors' perceptions of their patients' beliefs about therapeutic intent with the patients' actual beliefs about therapeutic intent, in the subgroup of 52 patients who were being treated radically. There were five patients who incorrectly believed they were being treated palliatively, but this misunderstanding was unrecognized by the doctor in four of these five cases. Figure 5b compares the doctors' perceptions of their patients' beliefs about therapeutic intent with patients' actual beliefs about therapeutic intent, in the subgroup of 48 patients undergoing palliative treatment. Sixteen patients incorrectly believed they were being treated for cure, but in 15 of these 16 cases the doctor did not realize that the patient misunderstood the situation.

*Reliability of observations*

Ten videotaped interviews, selected randomly, were reviewed by two additional observers who each completed the same abstraction sheet used by the interviewer. The information abstracted by each of the three observers was then compared. In these ten cases, there were no disagreements among the three observers about the patient's understanding of the diagnosis or of the treatment intent. In nine of the ten cases, all three observers concurred in their assessment of the patient's views of stage of disease. In one case, two observers said that the patient thought the disease was localized but the third observer was unsure. There was disagreement about the patient's expectation of cure in three cases. Two of these disagreements were minor: in one case, two observers said that the patient believed he had no chance of being cured, but the third observer said that the patient thought there was a slight chance of cure. In another case, two observers said that the patient believed the chance of cure was greater than 50%, but the other observer thought the patient was certain that he was curable. In the final case, one observer believed the patient was certain of being cured, while the other two observers were uncertain of the patient's views. There were no disagreements about the patient's perceptions of the probability of prolongation of life or about the probability of returning to normal activities after treatment.

In seven other cases, patients consented to a second full interview within two weeks of the first. There were no changes in their stated perceptions of diagnosis, stage of disease, treatment intent or probability of return to normal activities after treatment. There were minor changes in several patients' stated perceptions of the probability of cure. Two patients who had initially said that the chances of cure were greater than 90% were even more optimistic in the second interview and said they were certain of being cured. One patient, who said that he was certain of cure in the first interview, said in the second interview that his chance of cure was 99%. One final patient, who was unwilling to commit himself about the probability of cure in the first interview, stated in the second that he thought the chance of cure was greater than 50%.

Thus, there are only minor differences in the patients' answers to questions in interviews which are repeated after a short interval and there are few disagreements among different observers about their interpretation of the patient's views based on their observations of the interviews.

Doctors' perceptions

| Patients' perceptions | 100% | >50% | <50% | 0% | Uncertain | **Total** |
|---|---|---|---|---|---|---|
| 100% | 1 | 9 | 18 | 8 | 3 | 39 |
| >50% | 0 | 2 | 4 | 0 | 1 | 7 |
| <50% | 0 | 0 | 1 | 1 | 0 | 2 |
| 0% | 0 | 0 | 0 | 0 | 0 | 0 |
| Uncertain | 0 | 0 | 0 | 0 | 0 | 0 |
| **Total** | 1 | 11 | 23 | 9 | 4 | **48** |

**Figure 4** A comparison of doctors' perceptions of the probability of treatment prolonging the patients' lives with the patients' perceptions of the probability of treatment prolonging their lives.

Doctors' perception of patients' understanding

**a**

| Patients' perceptions | Cure | Palliation | Uncertain | **Total** |
|---|---|---|---|---|
| Cure | 38 | 0 | 9 | 47 |
| Palliation | 4 | 0 | 1 | 5 |
| Uncertain | 0 | 0 | 0 | 0 |
| **Total** | 42 | 0 | 10 | **52** |

**b**

| Patients' perceptions | Palliation | Cure | Uncertain | **Total** |
|---|---|---|---|---|
| Palliation | 27 | 0 | 4 | 31 |
| Cure | 9 | 1 | 6 | 16 |
| Uncertain | 1 | 0 | 0 | 1 |
| **Total** | 37 | 1 | 10 | **48** |

**Figure 5** (a) A comparison of doctors' perceptions of the patients' understanding with the patients' stated understanding of their prognosis, for the 52 curable patients. (b) A comparison of doctors' perceptions of the patients' understanding with the patients' stated understanding of their prognosis, for the 48 patients being treated palliatively.

### Discussion

Almost all the cancer patients in this series were aware of their diagnosis. Most patients with early stage, potentially curable cancers realized that the disease was localized and that they were being treated for cure. However, these curable patients were almost uniformly more optimistic about their prognosis than the treating physician. Patients with metastatic cancer often believed that the disease was localized and many incurable patients apparently failed to understand that they were being treated palliatively. Although some incurable patients recognized that their treatment would not cure them, almost all of them expected that treatment would prolong their lives.

Taken at face value, these observations imply that some patients do not have a sufficiently accurate understanding of their illness to become fully involved as partners with their physicians in making treatment decisions. An incurable patient who erroneously believes he has a high chance of cure is not in a position to perform the cost-benefit analysis which leads to an appropriate treatment decision in a palliative setting (Golden, 1970). Such patients are at risk of accepting, or even demanding, aggressive or toxic forms of treatment from which they may benefit very little. It can be argued that physicians recognize such patients and revert to their traditional paternalist role, making the appropriate decisions without input from the patient. Unfortunately, our data demonstrate that patients who seriously misunderstand their situation almost always pass unrecognized.

Unfortunately we did not make direct observations of interactions between doctors and patients and we can therefore only speculate as to how these misunderstandings arise. Patients' inaccurate views of their illness may simply be due to imperfect communication between the doctor and the patient. The physicians in this study believed that they had accurately described the extent of the disease and the goals of treatment to their patients, but Golden (1970) has previously demonstrated that doctors do not always communicate as well with their patients as they think they do and Reynolds *et al.* (1981) have shown that the way in which information is presented to cancer patients affects both their recall and their understanding. It appears from our data that an attempt was always made to discuss the disease honestly with the patient, but it is possible that the information given to patients with a poor prognosis may have been presented too optimistically.

It is also known that some patients who are seriously ill never fully acknowledge the gravity of their situation, no matter how well it is explained to them (Cassileth, 1980*b*). Taylor (1983) claims that this phenomenon of denial represents a normal adaptive process which permits the individual to cope with an otherwise unacceptable situation. Unfortunately, our data do not allow us to discern the relative importance of failed communication and/or denial in causing the misunderstandings we have observed.

The reliability of our observations is open to question, but repeat interviews carried out on seven patients who consented to a second interview, one to two weeks after the first, revealed no major changes in their responses. Furthermore, a random sample of the interviews has been reviewed by two other observers and there were no significant disparities in the observers' interpretations of the patients' views. It is recognized, however, that we have described the views of these patients at a single point in the evolution of their illnesses and that their perceptions may change over time. Our observations describe the practice of 10 oncologists in a single centre and it is possible that patients in other clinics may have a more accurate view of their illness than ours, although it seems more likely that these problems are widespread.

Modern medical ethics emphasize the patients' right to make their own decisions, but it has been pointed out that physicians have a continuing responsibility to ensure that these decisions are wise (Sider & Clements, 1985). Strull *et al.* (1984) have shown that doctors may overestimate their patients' desire to become actively involved in decisions about their care; here, we have demonstrated that doctors also overestimate their patients' understanding of their illness. 'Mutual participation' in medical decisions is a legitimate goal in the doctor-patient relationship, but it may not be what every patient wants and, unless communication improves, it is not what every patient needs.

Supported by grants from the National Cancer Institute of Canada and the Ontario Lung Association (WJM).

The authors wish to thank the ten oncologists at the Kingston Regional Cancer Centre for their assistance and cooperation. We also acknowledge the help of Ms Sandra Tirelli in the preparation of the manuscript.

### References

BRODY, D.S. (1980). The patient's role in clinical decision-making. *Ann. Int. Med.*, **93**, 718.

CASSILETH, B.R., ZUPKIS, R.V., SUTTON-SMITH, K. & MARCH, V. (1980*a*). Informed consent – Why are its goals imperfectly realized? *N. Engl. J. Med.*, **302**, 896.

CASSILETH, B.R., ZUPKIS, R.V., SUTTON-SMITH, K. & MARCH, V. (1980*b*). Information and participation preferences among cancer patients. *Ann. Int. Med.*, **92**, 832.

EPSTEIN, L. & LASAGNA, L. (1969). Obtaining informed consent. *Arch. Int. Med.*, **123**, 682.

FREID, C. (1974). Medical experimentation: Personal integrity and social policy. North-Holland Publishing Company: Amsterdam.

GOLDEN, J.S. & JOHNSTON, G.D. (1970). Problems of distortion in doctor-patient communications. *Psychiat. Med.*, **1**, 127.

HARDY, R.E., GREEN, D.R., JORDAN, H.E. & HARDY, G. (1980). Communication between cancer patients and physicians. *South. Med. J.*, **73**, 755.

HAUG, M.R. & SUSSMAN, M.B. (1969). Professional autonomy and the revolt of the client. *Soc. Prob.*, **17**, 153.

JENSEN, P.S. (1981). Doctor-patient relationship: Headed for impass or improvement? *Ann. Int. Med.*, **95**, 769.

MACKILLOP, W.J. & JOHNSTON, P.A. (1986). Ethical problems in clinical research: The need for empirical studies of the clinical trials process. *J. Chron. Dis.*, **39**, 177.

NOVACK, D.H., PLUMER, R., SMITH, R.L., OCHITILL, H., MORROW, G.R. & BENNETT, J.M. (1979). Changes in physicians' attitudes towards telling the cancer patient. *J.A.M.A.*, **241**, 897.

PARSONS, T. (1951). *The Social System*. The Free Press: New York.

REYNOLDS, P.M., SWANSON-FISHER, R.W., POOLE, A.D., HARKER, J. & BYRNE, J. (1981). Cancer and communication: Information-giving in an oncology clinic. *Br. Med. J.*, **282**, 1449.

ROBINSON, G. & MERAV, A. (1976). Informed consent: Recall by patients tested post-operatively. *Ann. Thorac. Surg.*, **22**, 209.

SIDER, R.C. & CLEMENTS, C.D. (1985). The new medical ethics: A second opinion. *Arch. Int. Med.*, **12**, 2169.

STRULL, W.M., LO, B. & CHARLES, G. (1984). Do patients want to participate in medical decision-making? *J.A.M.A.*, **252**, 2990.

SZASZ, T.S. & HOLLANDER, M.H. (1956). A contribution to the philosophy of medicine: The basic models of the doctor-patient relationship. *Arch. Int. Med.*, **9**, 585.

TAYLOR, S.E. (1983). Adjustment to threatening events: A theory of cognitive adaptation. *Am. Psych.*, **38**, 1161.